# Identification of reaction organization patterns that naturally cluster enzymatic transformations

**DOI:** 10.1186/s12918-018-0583-9

**Published:** 2018-05-30

**Authors:** Carlos Vazquez-Hernandez, Antonio Loza, Esteban Peguero-Sanchez, Lorenzo Segovia, Rosa-Maria Gutierrez-Rios

**Affiliations:** 1 Departamento de Microbiología Molecular, Instituto de Biotecnología Universidad Nacional Autónoma de México, Apdo, Postal 510-3, 62250 Cuernavaca, Morelos Mexico; 2 Departamento de Ingeniería Celular y Biocatálisis, Instituto de Biotecnología Universidad Nacional Autónoma de México, Apdo, Postal 510-3, 62250 Cuernavaca, Morelos Mexico

**Keywords:** Metabolic reaction, Reaction patterns, Compound transformation, Reactant pairs, Enzyme catalysis

## Abstract

**Background:**

Metabolic reactions are chemical transformations commonly catalyzed by enzymes. In recent years, the explosion of genomic data and individual experimental characterizations have contributed to the construction of databases and methodologies for the analysis of metabolic networks. Some methodologies based on graph theory organize compound networks into metabolic functional categories without preserving biochemical pathways. Other methods based on chemical group exchange and atom flow trace the conversion of substrates into products in detail, which is useful for inferring metabolic pathways.

**Methods:**

Here, we present a novel rule-based approach incorporating both methods that decomposes each reaction into architectures of compound pairs and loner compounds that can be organized into tree structures. We compared the tree structure-compound pairs to those reported in the KEGG-RPAIR dataset and obtained a match precision of 81%. The generated tree structures naturally clustered all reactions into general reaction patterns of compounds with similar chemical transformations. The match precision of each cluster was calculated and used to suggest reactant-pairs for which manual curation can be avoided because this is the main goal of the method. We evaluated catalytic processes in the clusters based on Enzyme Commission categories that revealed preferential use of enzyme classes.

**Conclusions:**

We demonstrate that the application of simple rules can enable the identification of reaction patterns reflecting metabolic reactions that transform substrates into products and the types of catalysis involved in these transformations. Our rule-based approach can be incorporated as the input in pathfinders or as a tool for the construction of reaction classifiers, indicating its usefulness for predicting enzyme catalysis.

**Electronic supplementary material:**

The online version of this article (10.1186/s12918-018-0583-9) contains supplementary material, which is available to authorized users.

## Background

One of the main forces defining genome content is metabolism, a chemical system that generates all the necessary chemical substances in living cells through chemical reactions mainly catalyzed by genome-encoded enzymes [1]. The increased availability of metabolic information has attracted the interest of bioinformaticians, who have developed computational methods to uncover important information regarding global properties of metabolic systems and detailed group reaction exchanges. However, the results arising from system-oriented studies are difficult to incorporate directly into the enzyme catalysis context.

Approaches based on graph theory build compound–compound networks that reveal hubs (compounds that are highly connected) and modules (compound sets that suggest communities of similar chemical and functional properties) [2]. Various properties arise from the removal of hubs, providing a partial view of the metabolic network due to loss of biochemical information required to include compound modifications present in more traditional metabolic maps. Such a level of detail can be achieved by adopting a reaction as a unit with the aim of analyzing specific compound transformations. Arita M. created a method to detail atom correspondence between substrates and products within reactions, demonstrating that metabolic networks do not follow a small-world network distribution (many elements in modules connected by a few hubs) [3].

Other methods used to describe relationships between chemical compounds that participate in metabolic reactions are so-called atom mappers, which automatically compare compounds to locate group transfer. An interesting property of atom mappers is that they identify structural transformations between single-compound pairs, allowing creation of reactant pairs (RPairs) such as those in the Kyoto Encyclopedia of Genes and Genomes (KEGG) RPAIR database, which are defined as “pairs of compounds that have atoms or atom groups in common on two sides of a reaction” [4]. RPairs have also been proven to be useful when combined with hierarchical clustering algorithms to elucidate relationships between reactions and enzymes [5], ultimately defining groups of compounds with related metabolic pathways [6]. These methods have been developed as promising alternatives for pathway discovery [7] but are slow to automate [8] and tend to require both a priori information and secondary methods to handle special cases, such as compounds with rings, prior to the final step of manual curation. Nonetheless, atom transfer is still a useful tool for predicting enzymatic catalysis in reaction sets [9]. Considering the aforementioned properties of atom mappers and graph theory as tools for pathway discovery, the first goal of our work was to implement a simple and fast method to complement atom mappers with the aim of avoiding a manual curation step as much as possible. For this purpose, we performed a statistical comparison of the TS-pairs proposed by our method with those in the RPAIR/RCLASS datasets, generating a precision value that can be interpreted as the confidence of the predicted set of reactant-pairs. We propose that the results obtained by our method will provide researchers with sets of confident pairs, a property proven to show better performance in pathfinders. Most importantly, our results can be used as a guide to determine which reactions should be candidates for manual curation.

Therefore, we systematically analyzed single-chemical metabolic reactions and outlined the mutual associations of their compounds, representing each reaction as a tree structure (TS). We then constructed a rule-based approach centered on two principles: (i) wide sets of metabolic reactions involve compounds, such as pool metabolites and cofactors, that undergo small modifications; and (ii) some compounds, usually coenzymes or pool metabolites, are frequently associated with various reactions in unrelated metabolic pathways. The first principle was used to create the *balance rule*, and the second was used to propose the *count rule*. Both rules were employed to analyze a set of 6230 well-curated reactions documented in the KEGG database. Each TS contained at least one pair of compounds similar in structure to RPairs. To assess the performance of our rules, we compared each resultant pair in a reaction to curated RPairs from the KEGG database (8) and obtained a precision value of 81% for the full set.

Because our approach outlines the specific architecture of each reaction, it can then be grouped according to its TS topology, grouping reactions of the same pattern into a single cluster of TSs (CTS). As a result, all the 6230 reactions analyzed clustered into 71 CTSs. Moreover, the reactions grouped in each CTS transfer similar chemical groups. Based on this property, we propose that our rule-based approach can be used as a classifier of metabolic reactions. Given this observation, we analyzed whether our CTSs were associated with enzyme categories represented by the Enzyme Commission number (ECN) available for each reaction [10]. Therefore, we measured the enrichment of each ECN in each CTS and revealed that the ECNs naturally fit the chemical patterns disclosed by our protocol.

## Results

### Generation of tree structures

**Fig. 1:**
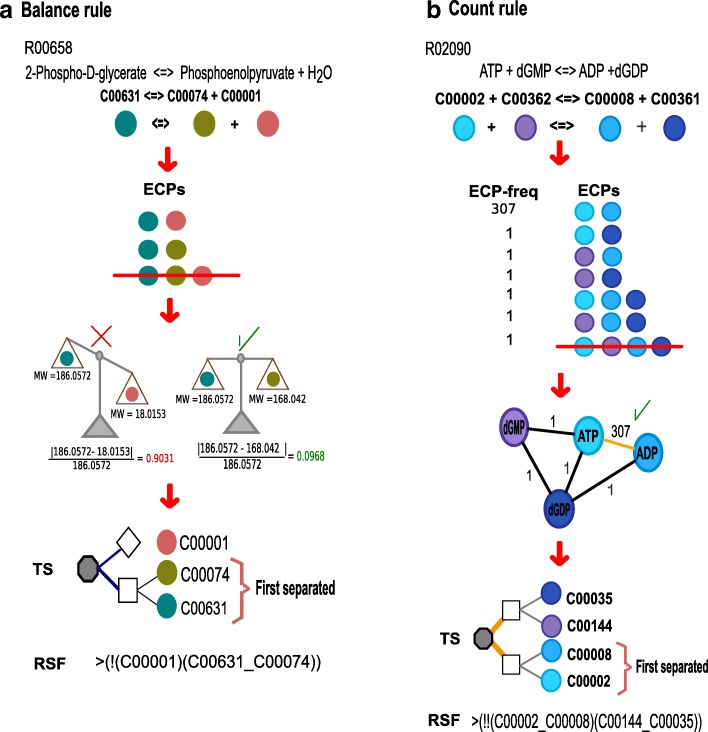
Representation of the constructed rules and tree structure (TS) construction. We illustrate graphical representations of our rules and their applications. **a** Example of using the *balance rule* to generate a tree structure for reaction R00658, in which the substrate 2-phospho-D-glycerate (C00631) is transformed into phosphoenolpyruvate (C00074) and water (C00001), by selecting the element of the Cartesian product (ECP) C00631_ C00074 as the element with the smallest difference in molecular weight within the reaction. **b** Example of using the *count rule* to construct a tree structure for reaction R02090, in which the substrates ATP (C00002) and dGMP (C00362) are transformed into ADP (C00008) and dGDP (C00361), by selecting the element of Cartesian product C00002_C00008 as the more frequently represented ECP in the entire network. For the TS representation, nodes: the gray octagon defines the tree root (reaction), the squares define the CP node, the rhomboid defines lone compound nodes, and the white circle defines compounds. Edges: blue, split through balance; orange, split through count; thin line, node/compound link. For the reaction string format (RSF), we represent the split in a line described in detail in the Methods section

Cofactors used in metabolic reactions are compounds that suffer small transformations compared to other reactants that are modified dramatically in one or more steps. One way to compare these changes within reactions is to measure the difference in molecular weights between compounds. Using this observation, we devised a computational protocol, called the “*balance rule*”, that estimates the similarity of compounds within a reaction. The purpose of this rule is to distinguish groups of compounds participating in a reaction based on differences in their molecular weights (Fig. 1a).

**Fig. 2:**
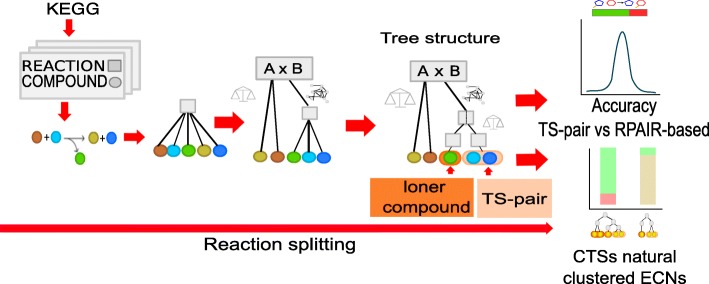
Analysis of the metabolic network using the rule-based approach. We illustrate the general procedures of how we applied the *balance* and *count rules* to the KEGG database. The procedure starts with the compounds and reactions from the KEGG, and the rules are applied to each reaction to generate a tree structure. Tree structures with identical architecture organizations are clustered together. Finally, we calculate the RPAIR correlation and Enzymatic Commission number (ECN) abundance levels to evaluate the performance of the method

As a first step, for each analyzed reaction, we assigned the elements of the Cartesian product (ECPs) derived from the compounds on the left and right sides of the equation, estimating the relative mass for each (described in more detail in the Methods section). The ECP with the smallest difference in relative mass was then separated from the rest of the reaction, and then all ECPs of the reaction for which the compounds were previously selected were eliminated (Fig. 1a). The remaining ECPs were then subjected to the same process until no ECPs remained or until the establishing of a difference between the relative ECP masses was not possible. Thus, at the end of the process, no compounds in the analyzed reaction participated in more than one ECP. The implications of this restriction are discussed in the next section. We represented rounds of separation and selection into a tree structure (TS) in which the tips showed the selected ECPs that described the pairs and loner compounds defining each reaction (Fig. 2). When applying the previous rules, we observed that for some reactions, the establishing of mass differences for particular sets of ECPs was not possible. For these reactions, our protocol uses a second rule, called the “*count rule*”, that selects an ECP based on its frequency in the entire network (Fig. 1b). The notion behind this rule is that some compounds, such as coenzymes and pool metabolites, are frequently associated with numerous reactions in many unrelated pathways. This property has been used previously to identify metabolic network hubs [2]. In our workflow, the *balance rule* is always applied first because it reflects an internal reaction property, and the *count rule* is used when the *balance rule* fails to select a single ECP.

To validate our approach, we used only the 6392 reactions that were fully described in the KEGG LIGAND dataset that were available in 2015 (see Methods section) [11]. From the original set, we discarded 1099 reactions with just one substrate and one product, including 335 reactions cataloged as isomerizations, which occur when a substrate atom is arranged into another position on the product. For example, in reaction R01518, 2-phospho-D-glycerate (C00631) is transformed into 3-phospho-D-glycerate (C00197) by isomerization. For this reaction, the only possible tree structure would be formed by both compounds (C00631_C00197) alone, and we therefore decided not to include these transformations in successive analyses. The same splitting method was applied to the rest of the group, including 642 reactions lacking an enzyme entry.

**Fig. 3:**
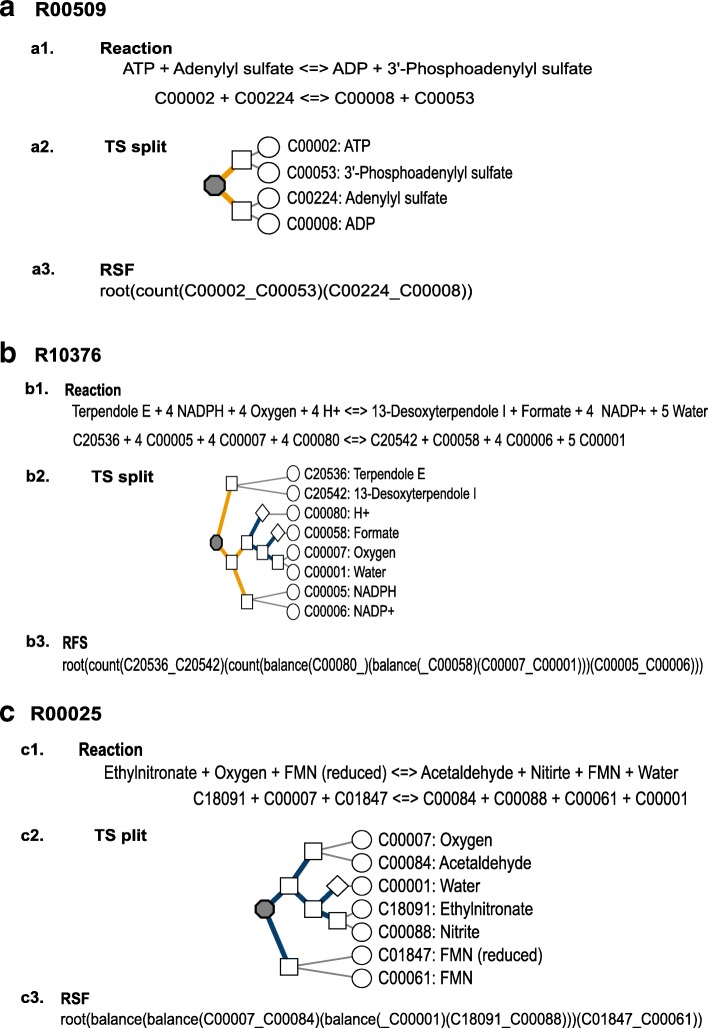
Representation of three tree structure examples. Panel **a** shows the split of reaction R00509 (subsection a1) as represented in the KEGG database. Subsection a.2 shows a graphical representation of a TS. The TS split representations are viewed from the top down, and compact strings are read left to right. Nodes: the gray octagon defines the tree root (reaction), the squares define the CP node, the rhomboid defines lone compound nodes, and the white circle defines compounds. Edges: blue, split through balance; orange, split through count; thin line, node/compound link. In subsection a.3, we represent the reaction split in a string format constructed as explained in the Methods section. In panels **b** and **c**, we show the TS graphical representation of reactions R10376 and R00025, respectively. For both reactions, the same caption descriptions for panels a2 and a3 are applied to each respective subsection

The *balance rule* was able to describe 6291 of 6392 reactions from the KEGG database, and the *count rule* was used for the 101 remaining reactions. Importantly, 15 of these 101 reactions, including reaction R00509, have a small number of reactants (one or two) that are transformed into one or two products (Fig. 3a). In this case, the compound arrangements were selected using only the *count rule*. The 86 remaining reactions that were involved in more complex transformations required both rules to generate the TS for each reaction. An example is presented in Fig. 3b for reaction R10376, which is involved in the biosynthesis of indole diterpene alkaloids. The selection begins by separating the terpendole E (C20536) and 13-desoxyterpendole I (C20542) molecules from the rest of the reaction to form the pair (C20536_C20542). The *count rule* was then used again to separate NADP+ (C00006) and NADPH (C00005) from the other compounds in the reaction to form the pair (C00005_ C00006). Finally, the minimal differences in molecular weight for the remaining compounds were sufficient to complete the process using the *balance rule*.

Another notable observation from our approach was that the TSs found for the 6392 reactions revealed common architectures that could be clustered considering the order of splitting within the reaction, and the two rules were used by the protocol. This clustering procedure resulted in a set of 71 different TS arrangements that included all the reactions tested (Table 1 and Additional file 1: Table S2). Notably, the tips of each TS were composed of two architecture types: pairs (two compounds associated directly through ECP selection by leaving exactly one compound on each side of the equation) and loner compounds (compounds from pair selection that remain alone on either side) (Fig. 2). A graphical representation of these architectures is shown in Additional file 1: Table S2. The next step was to validate the proposed architectures and interpret their chemical properties.

**Table 1:** Patterns identified and their condensed representations

TS-cluster identifier	CTS patterns	Number of reactions per CTS	Rule used to generate the pattern
CTS-1	>(!(C_C)(C_C))	1627	Balance
CTS-2	>(!(C)(C_C))	1059	Balance
CTS-3	>(!(C)(!(C)(C_C)))	1028	Balance
CTS-4	>(!(C)(!(C_C)(C_C)))	897	Balance
CTS-5	>(!(C)(!(!(C)(!(C)(C_C)))(C_C)))	439	Balance
CTS-6	>(!(C)(!(!(C)(C_C))(C_C)))	349	Balance
CTS-7	>(!(C_C)(!(C_C)(C_C)))	150	Balance
CTS-8	>(!(C_C)(!(C)(C_C)))	143	Balance
CTS-9	>(!(!(C)(C_C))(!(C)(C_C)))	103	Balance
CTS-10	>(!(C)(!(!(C_C)(C_C))(C_C)))	97	Balance
CTS-11	>(!(C)(!(!(C_C)(!(C)(C_C)))(C_C)))	82	Balance
CTS-12	>(!(C)(!(C)(!(C)(C_C))))	66	Balance
CTS-13	>(!(!(C)(C_C))(C_C))	54	Balance
CTS-14	>(!(C_C)(!(C)(!(C)(C_C))))	42	Balance
CTS-15	>(!(!(C)(!(C)(C_C)))(C_C))	29	Balance
CTS-16	>(!(!(C_C)(!(C)(C_C)))(C_C))	26	Balance
CTS-20	>(!(C)(!(C)(!(C)(!(C)(C_C)))))	12	Balance
CTS-21	>(!(C)(!(C_C)(!(C)(C_C))))	12	Balance
CTS-23	>(!(C_C)(!(C_C)(!(C)(C_C))))	8	Balance
CTS-24	>(!(C_C)(!(!(C)(C)_C))(C_C)))	8	Balance
CTS-25	>(!(C)(!(!(!(C)(C_C))(C_C))(C_C)))	5	Balance
CTS-26	>(!(!(C)(C_C))(!(C_C)(C_C)))	5	Balance
CTS-29	>(!(C)(!(!(C)(!(C)(!(C)(C_C))))(C_C)))	4	Balance
CTS-30	>(!(C)(!(!(C)(C_C))(!(C)(C_C))))	4	Balance
CTS-31	>(!(!(C_C)(C_C))(C_C))	4	Balance
CTS-33	>(!(C)(!(!(C)(!(C_C)(!(C)(!(C)(C_C)))))(C_C)))	3	Balance
CTS-34	>(!(C)(!(!(C)(!(!(C)(C_C))(C_C)))(C_C)))	3	Balance
CTS-38	>(!(C)(!(!(C)(C_C))(!(!(C)(C_C))(C_C))))	2	Balance
CTS-39	>(!(C)(!(C_C)(!(!(C)(C_C))(C_C))))	2	Balance
CTS-40	>(!(C)(!(!(C)(!(C)(C_C)))(!(C)(C_C))))	2	Balance
CTS-41	>(!(!(C)(C_C))(!(!(C)(C_C))(C_C)))	2	Balance
CTS-42	>(!(!(C)(!(C)(C_C)))(!(C)(!(C)(C_C))))	2	Balance
CTS-44	>(!(C)(!(C)(!(C)(!(C)(!(C)(C_C))))))	2	Balance
CTS-45	>(!(C_C)(!(C)(!(C_C)(C_C))))	2	Balance
CTS-46	>(!(!(C)(!(C)(C_C)))(!(C)(C_C)))	2	Balance
CTS-47	>(!(!(C_C)(C_C))(!(C)(C_C)))	2	Balance
CTS-50	>(!(!(C)(!(C)(C_C)))(!(C_C)(!(!(C)(C_C))(C_C))))	1	Balance
CTS-51	>(!(C)(!(!(C)(!(!(C)(C_C))(!(C)(C_C))))(C_C)))	1	Balance
CTS-52	>(!(!(C)(!(C)(C_C)))(!(C)(!(C)(!(C)(!(C)(C_C))))))	1	Balance
CTS-53	>(!(C)(!(!(C)(!(C)(!(C_C)(C_C))))(C_C)))	1	Balance
CTS-54	>(!(C)(!(!(C_C)(C_C))(!(C)(!(C)(C_C)))))	1	Balance
CTS-55	>(!(C_C)(!(!(C)(!(C)(C_C)))(!(C)(C_C))))	1	Balance
CTS-57	>(!(C)(!(C_C)(!(C)(!(C_C)(C_C)))))	1	Balance
CTS-58	>(!(C)(!(!(C)(!(C_C)(C_C)))(C_C)))	1	Balance
CTS-59	>(!(C_C)(!(C)(!(C_C)(!(C)(C_C)))))	1	Balance
CTS-60	>(!(C_C)(!(C_C)(!(C)(!(C)(C_C)))))	1	Balance
CTS-61	>(!(C_C)(!(C_C)(!(C_C)(C_C))))	1	Balance
CTS-62	>(!(!(C)(C_C))(!(C)(!(C_C)(C_C))))	1	Balance
CTS-65	>(!(!(C)(C_C))(!(C)(!(C)(C_C))))	1	Balance
CTS-66	>(!(!(C)(!(C)(!(C)(C_C))))(C_C))	1	Balance
CTS-19	>(!!(C_C)(C_C))	14	Count
CTS-71	>(!!(C)(C_C))	1	Count
CTS-17	>(!!(!(C)(C_C))(!(C_C)(C_C)))	24	Count-Balance
CTS-18	>(!!(!(C_C)(C_C))(!(C)(!(C)(C_C))))	16	Count-Balance
CTS-22	>(!!(C_C)(!(C)(C_C)))	10	Count-Balance
CTS-27	>(!!(!(C)(C_C))(!(C)(!(C)(C_C))))	5	Count-Balance
CTS-28	>(!(C)(!!(C)(C_C)))	5	Count-Balance
CTS-32	>(!!(!(C)(C_C))(!(C)(C_C)))	4	Count-Balance
CTS-35	>(!!(!(C)(!(C)(C_C)))(!(C)(!(C)(C_C))))	3	Count-Balance
CTS-36	>(!!(!(C_C)(C_C))(C_C))	3	Count-Balance
CTS-37	>(!!(!(C)(C_C))(C_C))	3	Count-Balance
CTS-43	>(!!(!(C)(C_C))(!(!(C)(C_C))(C_C)))	2	Count-Balance
CTS-48	>(!!(C_C)(!(C)(!(C)(C_C))))	2	Count-Balance
CTS-49	>(!!(!(C)(!(C)(C_C)))(C_C))	2	Count-Balance
CTS-56	>(!!(!(C)(!(C_C)(C_C)))(!(C)(!(C)(C_C))))	1	Count-Balance
CTS-63	>(!!(C_C)(!!(!(C)(!(C)(C_C)))(C_C)))	1	Count-Balance
CTS-64	>(!!(!(C_C)(!(C)(C_C)))(!(C)(C_C)))	1	Count-Balance
CTS-67	>(!!(C_C)(!(!(C)(C_C))(C_C)))	1	Count-Balance
CTS-68	>(!!(!(C_C)(!(C)(C_C)))(C_C))	1	Count-Balance
CTS-69	>(!!(C_C)(!(C_C)(C_C)))	1	Count-Balance
CTS-70	>(!(C)(!!(C_C)(C_C)))	1	Count-Balance

We describe the general topologies of the reactions found by our approach. Column 1 indicates the CTS identifier, and Column 2 shows the general pattern found for groups of reactions. “>” indicates the root of the tree structure; “!” indicates use of the *balance rule*; “!!” indicates use of the *count rule*; and “C” indicates a compound. A pair is described as (C_C), and a loner compound is (C). The number of parentheses around the pair or loner compound indicates its depth in the tree and the number of partitions employed for separation. Column 4 describes the rule or rules used to generate the arrangements

### TS-compound pairs correlated to KEGG RPairs

**Fig. 4:**
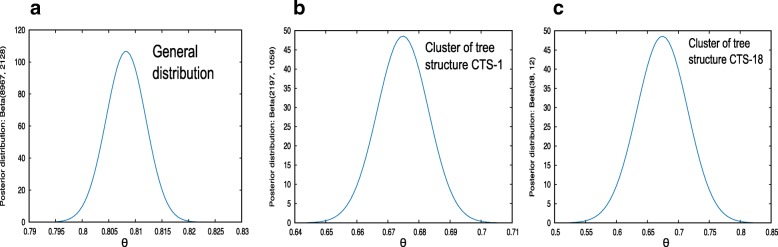
Correlation between TS pairs and RPAIRs. We present three examples of posterior distributions of the inferred parameter θ. **a** Total TS pairs vs. the entire RPAIR dataset. **b** Example showing the sharp-peaked distribution observed in CTS-1. **c** Example of a broad distribution showing the effect of a high correlation level in a group with a small number of reactions

We considered the presence of compound pairs identified for each reaction to evaluate the architecture of each TS. Compound pairs are also described in reactions as reactant pairs in the KEGG RPAIR database, in which a pair in a reaction represents group transfer interactions among compounds [4, 12]. The current version of the KEGG is different from previous versions in that RPAIR has been replaced by the RCLASS set, which contains only well-curated pairs according to the authors. Well-curated pairs are the “classification of reactions based on the chemical structure transformation patterns of substrate-product pairs (reactant pairs), which are represented by so-called RDM patterns” [11]. The 2015 version of the KEGG contains the original RPAIR, in which RCLASS pairs and other pairs have weaker evidence of being correct. We compared the RCLASS list to our predicted pairs reaction by reaction to measure the level of correspondence. From a total of 11,093 RPairs in the 6392 reactions, 8966 of our tree structure-compound pairs (TS-compound pairs) matched an RPair. We used a Bayesian approach to infer the probability of a TS-compound pair associating with its corresponding RPair to measure our prediction confidence levels. As extensively described in the Methods section, the confidence interval was determined by the 2.5 and 97.5% quantiles. Therefore, we found that the correspondence of the TS-compound pairs over the entire RCLASS set was 0.81, a value that should be interpreted as the precision of our method (Fig. 4a and Table 2) and that we consider very good given the simplicity of the method. Notably, some RPairs were constructed by mating one compound with more than one of the same reaction because an atom type can be shared by more than one compound, which is the case in the KEGG because a total of 1157 KEGG reactions are conformed by pairs sharing a common compound. This is an important difference between our approach and the manner in which atom mappers construct reactant pairs. An example showing the result of this difference is reaction R00025, where ethylnitronate (C18091) is catalyzed to nitrite (C00088) and acetaldehyde (C00084) by the enzyme nitronate monooxygenase (Fig. 3c). In this reaction, ethylnitronate is decomposed into three reactants pairs, RC00126-(C00061_C01847), RC02541-(C00084_C18091) and RC02759-(C00088_C18091), which correspond to (FMN_reduced_FMN), (acetaldehyde_ethylnitronate) and (nitrite_ethylnitronate), respectively. In this reaction, our protocol positively detected FMN_ reduced_FMN as a pair (C00061_C01847) and an additional group ((_C00001) (C00088_ C18091)) composed of water as a loner compound (_C00001) and the pair ethylnitronate_nitrite (C00088_ C18091) matching an RPair in RCLASS RC02759. Notably, water (C00001) is not associated with any RPair for reaction R00025. As an advantage, our method keeps this compound in the architecture and thus shows its contribution to the reaction. Because our method generates unique compound combinations as the final result, the undetected pair composed of acetaldehyde and ethylnitronate (RC02541-(C00084_C18091)) was eliminated as a pair at some other point in the process due to its presence in another ECP with a bigger mass difference compared with the remaining products in the tips of the tree. We also observed that our protocol generated 11,093 TS-compound pairs and from these 2127 are absent in the RPAIR data set, making these pairs candidates for manual curation to establish their confidence.

**Table 2:** Comparison of TS-compound pairs versus RPairs

Cluster ID	Number of total reactions	Number of reactions with only matching pairs	Number of reactions with no matched pairs	Number of reactions with mixed pair matches	Number of RPAIRs in a CTS	Number of matching TS-compound pairs	Number of mismatched TS-compound pairs	Inferior interval 0.025	Superior interval 0.975	Average value of the interval
General	6392	4869	618	905	11,193	8966	2127	0.8006	0.8153	0.8070
CTS-1	1627	960	391	276	3254	2196	1058	0.6587	0.6909	0.6748
CTS-2	1059	1016	43	0	1059	1016	43	0.9467	0.9704	0.9586
CTS-3	1028	926	102	0	1028	926	102	0.8818	0.9183	0.9
CTS-4	897	867	8	22	1794	1756	38	0.9717	0.985	0.9783
CTS-5	439	435	1	3	878	873	5	0.9884	0.9981	0.9933
CTS-6	349	341	0	8	698	690	8	0.9794	0.995	0.9872
CTS-7	150	1	17	132	450	206	244	0.412	0.5039	0.458
CTS-8	143	57	7	79	286	193	93	0.6195	0.7278	0.6737
CTS-9	103	51	3	49	206	151	55	0.6707	0.791	0.7309
CTS-10	97	2	0	95	291	196	95	0.6187	0.7261	0.6724
CTS-11	82	0	0	82	246	163	83	0.6024	0.7203	0.6613
CTS-12	66	58	8	0	66	58	8	0.7906	0.9453	0.868
CTS-13	54	39	7	8	108	86	22	0.7158	0.8664	0.7911
CTS-14	42	21	5	16	84	58	26	0.5882	0.7841	0.6862
CTS-15	29	28	0	1	58	57	1	0.9373	0.9996	0.9684
CTS-16	26	0	0	26	78	29	49	0.2687	0.4812	0.3749
CTS-17	24	0	0	24	72	48	24	0.5545	0.77	0.6623
CTS-18	16	8	0	8	48	37	11	0.6434	0.877	0.7602
CTS-19	14	5	6	3	28	13	15	0.2867	0.6467	0.4667
CTS-20	12	11	1	0	12	11	1	0.7151	0.9977	0.8564
CTS-21	12	4	3	5	24	13	11	0.3449	0.7318	0.5384
CTS-22	10	6	1	3	20	15	5	0.5443	0.9085	0.7264

We compared the numbers of hits and fails of our predicted pairs with RPairs in the KEGG RPAIR dataset. The table presents precision estimations based on the calculated posterior probabilities of each group. The confidence interval and average value are shown in the last 3 columns

Because our protocol naturally clustered each reaction according to its architectural arrangement into 71 groups, we estimated the precision of CTSs for which the number of reactions was sufficiently large to perform a reasonable statistical inference. Therefore, estimations were performed only for CTSs with at least 10 reactions with predicted pairs. The results of our precision analysis of 6291 reactions clustered in 22 different CTSs are shown in Table 2, with the CTSs numbered from 1 to 22 (1 represents the group with the highest number of reactions, and 22 represents the group with the smallest number of reactions). As expected from the group size differences, the results presented in Table 2 show that the CTSs had different levels of precision. We observed eight CTSs encompassing 3879 reactions with an average confidence value higher than 0.80. For example, for CTS-5, we predicted only five pairs that were different from those assigned by the KEGG out of 878 total reactions (Table 2), indicating that every pair found in this CTS had a very high probability of being an RPair. We also noticed that all the reactions in CTS-5 were oxidoreductions reactions in which the incorporated oxygen is not necessarily derived from molecular oxygen.

Another 2198 reactions were grouped in CTSs with average precision values ranging from 0.79 to 0.60 (Table 2). For example, CTS-1 had the highest number of members with the same architecture arrangement (1627 reactions). From the 3254 possible pairs in this CTS, we predicted that 2196 matched an RPair, representing two-thirds of the group. This gave CTS-1 an average probability of 0.67, which also showed a sharp-peaked distribution (Fig. 4b). Therefore, this CTS provided a large amount of information supporting our conclusion that two of its three pairs are dependable RPairs, and the remaining one-third of the group (1058) represents candidates for validation through manual curation. We found that other CTSs in the same confidence range showed broad distributions, such as CTS-18 (Fig. 4c), which exhibited a value of 0.64 for the 2.5% interval and 0.87 for the 97.5% interval. In this case, in which 37 of 48 possible RPairs were matched, we had less information supporting our estimate, and the greater uncertainty could be explained by the small sample size (16 reactions). Despite the small sample size, the proportion of hits revealed that CTS-18 had an intermediate probability of yielding a positive RPair. Nevertheless, inspection of this CTS showed that all the reactions in the cluster are catalyzed by ligases classified by enzyme commissions in classes 6.3.4 and 6.3.5. Furthermore, two reactions involved in the biosynthesis of neomycin, kanamycin and gentamicin, R10767 and R10768, are catalyzed by a tobramycin carbamoyltransferase of the group 6.1.2.2, indicating that despite the medium significance, compounds of reactions in CTS-18 undergo similar transformations catalyzed by similar enzymes. The next step was to determine whether the reaction groups in the remaining CTSs also exhibit similar chemical transformations.

### Tree structure distribution reveals that the predicted patterns yield reaction groups with similar chemical events

The 2015 version of the KEGG retained the classification of RPairs proposed by Kotera M. et al. regarding “main-pairs (describing main changes in substrates), cofac pairs (describing changes in cofactors for oxidoreductases), trans pairs (focused on transferred groups for transferases), ligase pairs (describing the consumption of nucleoside triphosphates for ligases), and leave pairs (describing the separation or addition of inorganic compounds for enzymes, such as lyases and hydrolases)” [4]. These classes link compound pairs within a reaction with the enzyme activity exerted upon them, indicating that the same pair could be assigned to a different class depending on the reaction in which it participates. Therefore, our next step was to analyze our predicted pairs in this pair-reaction context. We labeled each pair with its RPAIR class (main, cofac, trans, leave or ligase), and we also labeled each pair according to the proportion of predicted RPairs in its corresponding reaction, resulting in four categories. The first category included reactions in which the predicted pairs (EPP) entirely fit the published RPairs. The second and third categories involved reactions with at least one hit pair and one or more failed pairs. We labeled these reactions as mixed pairs (MP) as they produced mixed-positive pairs (MPP) and mixed-failed pairs (MFP). The last group consisted of pairs in reactions in which no RPair was found, called failed pairs (FP). We labeled the pairs using three confidence ranges taken from the Bayesian probability estimation previously defined for their respective CTSs. The CTSs were ranked by their mean probability *x*, where trees with ¨high confidence¨ were in the range *x* ≥ 0.8, trees with “medium confidence” were between 0.8 > *x* ≥ 0.6, and CTSs in which 0.6 > *x* were labeled “low confidence”.

**Fig. 5:**
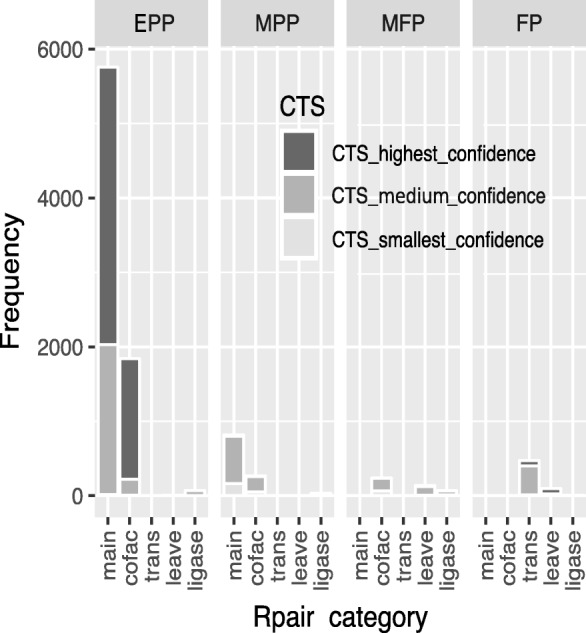
Analysis of TS pairs using RPAIR classes. The figure represents the abundance of each TS pair as a function of the class proposed by Kotera [4]. We categorized the pairs as being in reactions in which the whole predicted pair entirely fit the published RPair (we called this EPP). The second group includes reactions representing at least one hit with one or more fails, which we called mixed pairs, and this group was subdivided into positive (MPP) and failed pairs (MFP). The last group contains reactions in which we failed to detect an RPair, called failed pairs (FP). We also arranged the TS pairs according to their mean precision levels (x) estimated from the CTSs by Bayesian analysis. TS pairs in the precision range x ≥ 0.8 were classified as “high confidence”, those in the range 0.80 > x ≥ 0.60 were classified as “medium confidence”, and those in the range x < 0.60 were labeled “low confidence”

Figure 5 shows the frequency of the reactant pairs grouped according to their RPAIR class (main, cofac, trans, leave and ligase), our hit-fail categories (EPP, MPP, MFP and FP) and our confidence ranges (high, medium and low). The EPP category included a total of 7672 predicted pairs, and notably, 48% of these matched a main RPair with high confidence. Interestingly, another 21% of the EPP pairs fell in the cofactor class (*cofac*) with high confidence. A very small group, less than 1% of the EPP category, belonged to the *ligase* and *leave* classes, exhibiting a very small proportion of pairs with one and four members, respectively. In the same category, the other 26% of the pairs with medium confidence were main pairs, and another 2.7% were *cofac* pairs. Similar behavior was observed for the MPP category because the *main* and *cofac* categories predominated in 1120 pairs; however, in contrast to the EPP category, the concordant pairs exhibited mainly a medium confidence level. Another 240 pairs in the low-confidence group were mostly distributed in the *ligase* category. Pairs in the MFP group (446 pairs) tended to belong to the *cofac* and *leave* categories, both with medium confidence. Finally, as shown in Fig. 5, the approach failed to provide a concordant RPair for 576 reactions in the FP category. Interestingly, these FP pairs were classified by Kotera as *trans* and leave pairs, and 383 were clustered into CTS-1 (data not shown). Furthermore, they were shown to be preferentially catalyzed by hexosyltransferases, pentosyltransferases and phosphotransferases, with an alcohol group as the acceptor, and by some enzymes other than methyl groups that transfer alkyl or aryl groups.

**Fig. 6:**
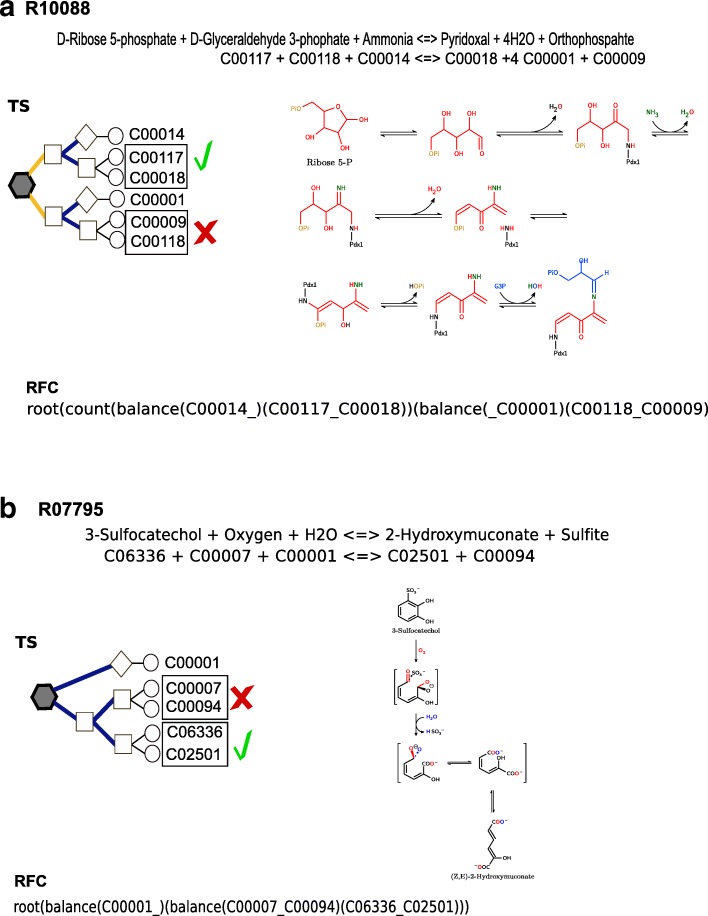
Manual curation of reactant-pairs. Panel **a** illustrates the proposed mechanism for reaction R00018 and its tree structure (TS). In this reaction, D-Ribose 5-phosphate (red) and D-Glyceraldehyde 3-phosphate (blue) are converted into Pyridoxal phosphate in the presence of an ammonia molecule (green). Figure adapted from reference [13]. Panel **b** illustrates the proposed mechanism for reaction R07795 and its TS. This reaction is the conversion of 3-Sulfocatechol into 2-Hydroxymuconate. Figure adapted from reference [14]. The pairs in TSs that follow that reaction’s proposed mechanism are marked by a checkmark. The TSs are also shown in string format (RSF), (see Methods)

The results presented thus far provide evidence that our rules are exceptional in defining pairs in reactions catalyzed by oxidoreductases. In contrast, these rules have limitations in establishing the transitions of specific types of transferases, lyases and hydrolases. The results obtained for these reactions prompted us to perform manual curation with the aim of establishing the degree to which the mechanisms proposed in the literature follow our proposed splitting when a statistical description could not be provided or when we obtained results that were inconsistent with the proposed RPairs. The first example is reaction R10088 from CTS-32 in which we obtained mixed pairs. As shown in Fig. 6a, this reaction entry describes the conversion of D-Ribose 5-phosphate and D-Glyceraldehyde 3-phosphate (G3P) into Pyridoxal phosphate (vitamin B6) in the presence of an ammonia molecule, yielding four molecules of water and inorganic phosphate (Pi). Our resulting TS shows that vitamin B6 is paired to the D-ribose 5-phosphate molecule (C00117_C00018) as proposed in the RCLASS ID RC03049 (C00018_C00117). The overall TS partially reflects the proposed mechanism, as shown in Fig. 6a. The vitamin is proposed to be assembled from ribose, ammonia and glyceraldehyde 3-phosphate (G3P), with the phosphate group from the ribose molecule leaving the complex [13]. In the TS, vitamin B6 is paired with the ribose molecule, with ammonia as the closest loner compound, which is consistent with the overall mechanism up to the intermediary before the excision of Pi. G3P (C00118), however, is paired with Pi (C00009) because they are more similar in terms of molecular weight, implying that the Pi group that enters and leaves is the group in G3P, not the group in the ribose molecule. The mapping proposed by the KEGG also pairs G3P with pyridoxal phosphate - RC01783 (C00018_C00118) -, which is consistent with the proposed mechanism [13]. Nonetheless, we should remember that our method does not consider molecular mechanisms in performing the splitting in contrast to atom mappers, which attempt to reflect the atom transitions between chemical species in detail. Finally, an important trait of the TS layout is the first splitting performed by the count rule, which reflects the fact that most of the mass in the pyridoxal molecule is already present in the intermediary prior to Pi excision. One important aspect of the reaction described in Fig. 6a is that the enzyme Pdx1 (pyridoxal 5′-phosphate synthase, ECN 4.3.3.6) prefers the substrate G3P and joins the molecule through imine formation, which is an important trait for understanding the mechanism and reaching the proposed conclusion. We think that considering enzymatic mechanisms will be valuable for improving our method.

Another interesting example, shown in Fig. 6b, is R07795 in CTS-4. The reaction describes the conversion of 3-sulfocatechol (C06336) into 2-hydroxymuconate (C02501). This reaction is catalyzed by the enzyme catechol 2,3-dioxygenase (ECN 1.13.11.2), which also catalyzes R04089 - the conversion of 2,3-dihydroxytoluene into 2-hydroxy-6-keto-2,4-heptadienoate - and reactions R05295, R05404 and R05406, all of which are implicated in conversions of different chemical species of catechol involving aromatic biodegradation. All these reactions, except for R07795, were clustered in CTS-2, which had a mean precision of 95%. Notably, the TS-pairs in these reactions match the reactions’ RCLASS pairs, which is important because these reactions yield an additional product despite the similarity in their ring cleavage mechanism by incorporating oxygen during biological oxidations of these organic substrates [14]. In contrast, in R07795, oxygen is incorporated into 3-sulfocatechol through an attack that releases sulfite, which we paired with oxygen (C00007_C00094). The 2015 version of RPAIR included this pair as a leave pair, but RCLASS ignores it due to mechanistic inconsistency. The mechanism proposed by Junker F et al. [14] is consistent with the KEGG data, and we also propose that this pair should be considered incorrect.

A count of the pairs recovered in the CTSs that were not statistically evaluated also reveals some patterns. Overall, 194 of the 324 pairs in these reactions are confirmed RCLASS entries. However, the proportion of pair matches is not homogeneous, which is similar to the 22 evaluated CTSs. For example, in CTS-32, which includes four reactions, seven of eight pairs are confirmed RCLASS entries.

The results presented in this section, determined using the Kotera classification, suggest that reactions in the CTSs might be correlated to specific catalytic conversions. We tested this hypothesis with the aim of proving whether our rules naturally cluster reactions that share the same ECN within a CTS because this number is used to classify reactions in terms of the enzymes by which they are catalyzed.

### Tree structures cluster into general enzyme patterns

**Fig. 7:**
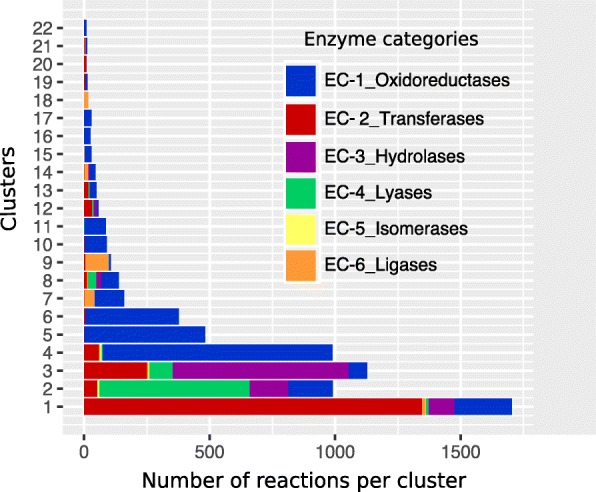
Correlation of tree structure clusters with general enzymatic categories. The clusters of tree structures (CTSs) tended to naturally group enzymatic categories provided by the Enzyme Commission numbers (ECNs). This figure presents this tendency using the first digit of the ECN class, in which enzymes are classified as oxidoreductases (ECN-1), transferases (ECN-2), hydrolases (ECN-3), lyases (ECN-4), isomerases (ECN-5) and ligases (ECN-6)

Analysis of our TS-compound pairs and CTSs suggested that our approach can cluster reactions catalyzed by enzymes belonging to similar classes as observed for the ligases of CTS-18. To evaluate the generality of this observation, we associated the ECNs corresponding to each reaction. ECNs are composed of four digits separated by periods, and the numbers represent a progressively specific classification of each enzyme. The first digit groups enzymes into general types of catalysis. For example, ECN type 1 (ECN-1) describes oxidoreductases that catalyze oxidoreduction reactions in which hydrogen and oxygen atoms or electrons are transferred from one substance to another. The subsequent digits add levels of specificity; ECN-1.1, for example, contains oxidoreductases that act on donor alcohol groups. Figure 7 shows the frequency of each ECN associated with each reaction grouped in each CTS considering only its first digit. The bar plot in this figure shows that all CTSs were concentrated mainly in the ECNs of a single class, and CTSs with higher numbers of members tended to include other ECNs of other classes in lesser proportions. This result can be explained by the fact that some reactions are catalyzed by more than one enzyme or by multifunctional enzymes. For example, CTS-2 has reactions catalyzed by the enzyme chloromuconate cycloisomerase (EC 5.5.1.7), which is cataloged as both an isomerase and an intramolecular lyase due to its capacity to release free hydrogen chloride when 2-chloro-cis, cis-muconic acid is transformed into cis-4-carboxymethylenebut-2-en-4-olide [15]. Therefore, finding a representation of more than one enzyme with similar catalysis types despite classification in different ECNs should not difficult.

As previously mentioned, isomerization reactions with only one substrate and one product were not considered because they form trivial tree structures. Nonetheless, isomerizations embedded in reactions with more than one compound on each side of the equation were considered and mapped to specific CTSs (CTS-2, CTS-3 and CTS-4). Similar distributions were observed for other ECNs, such as ligases represented in CTS-7, CTS-9, CTS-14 and CTS-18 and hydrolases represented in CTS-1, CTS-2 and CTS-3.

**Fig. 8:**
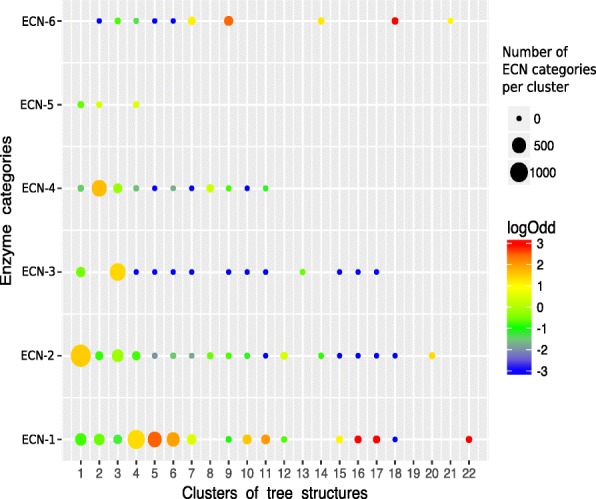
Enrichment of enzyme categories into tree clusters. We illustrate the results of the significance of each enzyme category within the clusters of tree structures (CTSs). The graph shows the logarithm of the odds ratio, which represents the strength of the enrichment (spectrum color bar), and the number of hits in the group (point density). We only show CTSs with False discovery rate < 0.05

As shown in Fig. 7, our CTSs tended to concentrate particular ECNs. To test whether this over-representation was statistically significant, we calculated the enrichment of each ECN in each CTS considering only the first ECN digit and CTSs grouping more than 10 reactions using Fisher’s exact test. We considered enriched CTSs to have *P values* < 0.05. Figure 8 shows the significantly enriched groups represented in terms of logarithmic odd ratios, which are useful for evaluating the strength of enrichment. The CTSs considered enriched in an ECN category were those displaying a false discovery rate, reported as the log*odd ratios* ≥ 0.5.

As shown in Figs. 7 and 8, the probability of having reactions catalyzed by specific enzyme types increased as the number of reactions in the CTS decreased, as shown by the significant enrichment in transferases, ligases and oxidoreductases in CTS-20, CTS-21 and CTS-22, respectively. Moreover, 17 of the 22 CTSs were significantly enriched in only one ECN, and another three were significantly enriched in only two ECNs; CTS-2 has lyases (ECN-4) and isomerases (ECN-5), CTS-4 has oxidoreductases (ECN-1) and isomerases (ECN-5), and CTS-7 has oxidoreductases (ECN-1) and ligases (ECN-6). An analysis of CTS-2, which is significantly enriched in ECN-5 and ECN-4, showed that its isomerases belong to the subclasses 5.5.1.7 (a chloromuconate cycloisomerase) and 5.5.1.11 (a dichloromuconate cycloisomerase). These enzymes catalyze eight reactions for the degradation of chlorocyclohexane or chlorobenzene, respectively, and they release a hydrogen chloride molecule in all cases. Surprisingly, when analyzing the KEGG database in more detail, we found two other reactions (R08119 and R09215) catalyzed by a chloromuconate cycloisomerase that were not grouped in CTS-2. Interestingly, the isomerization processes in R08119, which transforms 2-fluoro-cis, cis-muconate into 5-fluoromuconolactone, and R09215, in which 3,5-dichloro-2-methylmuconate is transformed into 3,5-dichloro-2-methylmuconolactone, do not yield halogens as reaction products because fluorine and chlorine remain bound to the products, respectively. This is a very good example showing that our rule-based approach can separate very similar reactions in CTSs with very high precision levels (mean 0.95, see Table 2) even if they are catalyzed by the same enzyme.

Figure 8 shows that of the 17 significantly enriched CTSs, the clusters CTS-16, CTS-17, CTS-18 and CTS-22 were most significantly enriched for specific ECN categories. Other groups, such as CTS-5 and CTS-9, were almost homogenous because they had only one or two reactions with ECNs of another class. Figure 8 shows that despite significant enrichment in only one ECN class at the first digit, CTS-20 did not have one of the highest odd ratios due to the small size of the group, which had only 10 elements. In contrast, CTS-5 clustered 482 reactions, 480 of which were classified in ECN-5.

Careful examination of the 17 CTSs with statistically significant enrichment in one ECN revealed a tendency to concentrate reactions with similar catalysis types, even at the second digit as shown for CTS-2. Other examples include reactions in CTS-17 catalyzed by enzymes of subclass 1.4.1 and involved in amino acid oxi-reductions. Reaction R00025 served as the exception because it is catalyzed by nitronate monooxygenase (1.13.12.16). Driven by this observation, we applied Fisher’s exact test while considering the second and third ECN digits, and our results showed that at these class levels, the remaining CTSs, including CTS-2 and CTS-17, tended to be significantly enriched in more specific ECNs. The results of these tests are shown in Additional file 1: Figures S1 and S2.

Considering the presented results, we conclude that our proposed rules showed that a significant number of the reactions evaluated reflected a clear biochemical link between tree structures and specific chemical events.

## Discussion

We described a clustering analysis of enzymatic reactions described in the KEGG database using our rule-based approach. Our results allowed us to classify different metabolic reactions into patterns, revealing associations among compounds at a glance. The rules of our approach were implemented as a protocol that first locates and separates groups with the smallest differences among compounds within a reaction. Thus, using our approach, the most different compounds are identified by successive elimination. The logic behind this approach is that some compounds, such as coenzymes and pool compounds (such as *S*-adenosyl-L-methionine [16]), tend to undergo less dramatic changes during a reaction (the basis of the *balance rule*) and tend to be associated with more enzymes in many unrelated pathways (the basis of the *count rule*). Other methods, such as compound mappers, highlight atom exchanges between substrates and products through heuristic graph-matching algorithms, which requires knowledge of the reaction modifications. Water, for example, is removed from the analysis to increase the detection of oxygen and hydrogen traces at the cost of losing catalysis conducted by hydrolysis [3, 12, 17]. In contrast, our approach does not remove any compound unless it is present in an unbalanced reaction or in a reaction in which the subscripts are not well defined, such as reaction R00001 (polyphosphate + n H_2_O < => (n + 1) oligophosphate). Therefore, we were able to analyze a wider range of compounds within reactions.

Our analysis also showed that despite the complexity of enzyme catalysis, reactions with similar catalytic patterns were grouped through our TSs, which outlined the chemical transformations of substrates and products within reactions. We demonstrated that the TSs preserved pair architectures comparable to those of reactant pairs of the RPAIR dataset [12], which were based on identification of the atom type [8]. In contrast, our approach based on two simple rules performed well, particularly for pairs classified in the RPAIR set as *main*, *cofac* (redox cofactors) and *ligases* (mainly phosphorylations). Our protocol was less precise when describing small groups of pairs cataloged as *trans* and *leave*, specifically transferring compounds such as hexose, pentose, alkyl or aryl groups and a small group of phosphotransferases. These reactions were found mainly in CTS-1 and CTS-2; therefore, different rules need to be established or a manual curation step should be implemented to verify these reactions.

We also found that for a particular set of reactions, TS pairs were predicted that did not correspond to the predicted RPairs, most of which were catalogued by Kotera as *trans* or *leave* [4]. R06726 (retronecine + 2 L-isoleucine <= > senecionine, grouped in CTS-2) serves as a good example of a reaction with inconsistent pairs. In this case, we predicted the pair (C0047_C06176, L-isoleucine_senecionine) by using a balance reaction transforming two L-isoleucine molecules. In comparison, the KEGG pairs a retronecine molecule with the senecionine in an RPair given that the molecules have more significant common atom types in contrast to L-isoleucine. Another five of six reactions in CTS-2 with a predicted pair according to our approach could not be compared due to the lack of an RPair. Three of these reactions (R07652, R07916 and R10177) were involved in geranyl and farnesyl transformations, which are catalyzed by enzymes that transfer acyl or aryl groups from the substrate to the product. Among these reactions, the ECPs defined as more similar included compounds with acyl or aryl groups because they have similar molecular weights and are separated by diphosphate, a compound with a smaller mass compared with that of the others within the reaction. These reactions in the KEGG were not assigned an RPair because they involve multiple steps. For this type of reaction, careful curation is required to evaluate the consistency of our partitioning.

We can conclude that our rules, which are based on intrinsic biochemical reaction properties and interactions of compounds across the whole network, organize reactions in individual tree structures, which is useful for clustering considering only the proposed compound arrangements. This approach is also helpful for easily identifying reactions with similar transformations. However, for a small group of transformations, this simple approach was insufficient, as shown in Fig. 5 for the MFP and FP categories. Therefore, this approach should be improved for these cases in future work.

Another advantage of our rule-based approach is that it does not eliminate any compounds during tree assembly, allowing observation of the relative ordering of compounds predicted by our rules within a reaction and among reactions, a feature that is not available in atom mappers.

Work published by Faust C. and coworkers [16] evaluated the effect of using well-curated reactant pairs and other parameters on reconstruction of metabolic pathways and showed that the metabolic network with the best performance combines biochemical knowledge encoded in the KEGG RPAIR with a weighting scheme that penalizes highly connected compounds. This is consistent with the pair-reaction classification that we tested, which shows that most of the our EPPs were main RPairs. We believe that incorporation of our rule-based approach into pathfinders will substantially improve the performance of these methods as our method could provide the relative relevance of compound pairs within reactions.

Kotera and coworkers [18] and Rahman et al. [9] developed methods to predict reactant pairs using similarity between reaction centers, proving the feasibility of grouping reactions by their ECNs through the chemical transformations of compounds. Our approach can cluster metabolic reactions by their general patterns, resulting in groups with significant enrichment in specific ECNs, even at the third digit. We found secondary ECN classes in smaller proportions of CTSs, which does not disprove the above observation because individual reactions can be linked to several ECNs. Furthermore, slight variations in these classes are expected due to factors such as multiple domain enzymes and enzyme promiscuity, which has been documented as a common phenomenon [19].

Statistical evaluation performed using the false discovery rate, represented as the odds ratio logarithm, showed that 10 of the 22 CTSs used in this analysis are enriched in oxidoreductases, which we also proved when testing the Kotera classification [4]. However, we discarded all reactions without an assigned ECN, and we predicted 49 CTSs that clustered 113 reactions that were not used in Fisher’s exact test. These 49 CTSs had less than ten reactions, and their small sample sizes made statistical tests less convenient. Therefore, the pair architectures found in these reactions are candidates for manual curation to test their reliability. Overall, we propose that with confirmation of our curated set, our CTSs can serve as good guides for predicting reaction catalysis.

## Conclusions

Some methodologies based on graph theory organize compound networks into metabolic functional categories without preserving biochemical pathways. Other methods based on chemical group exchange and atom flow trace the conversion of substrates into products in detail, which is useful for inferring metabolic pathways. To analyze metabolic networks, we presented a rule-based approach incorporating both methods that decomposes each reaction into architectures of compound pairs and loner compounds that can be organized into tree structures. We found that the tree structure-compound pairs fit those reported in the KEGG-RPAIR with excellent match precision. The generated tree structures naturally clustered all reactions into 71 general reaction patterns of compounds with similar chemical transformations. Finally, we evaluated the catalysis types in the clusters based on Enzyme Commission categories and revealed preferential use of enzyme classes. We demonstrated that applying simple rules can allow identification of reaction patterns reflecting metabolic reactions that transform substrates into products and the type of catalysis involved in the transformation. The pairs generated using our rule-based approach can be incorporated as inputs to improve the performance of pathfinders because well-curated pairs provided better results, as demonstrated by Faust and collaborators [7]. Our method is a reaction classifier that can correlate EC numbers to our CTSs. Therefore, we propose that our method could constitute a useful first step for the prediction of reaction catalysis, which can be conducted by simply incorporating the discarded reactions that were not considered in this analysis. Finally, using this last property, testing whether the enzymes clustered in the CTSs are evolutionarily related, will be useful, a task our group have started.

In future work, we intend to model chemical transitions of generic reactions, such as those with discordant pairs. For these, we will introduce fewer generic rules that will consider other measures different from molecular weight, such as giving carbon atoms a greater weight because carbon flow is the main feature of metabolic transformations. Additionally, as suggested by the results for the reactions described in terms of their catalytic mechanism, we will include the roles of enzymes in the reaction layout.

## Methods

### Datasets

We analyzed datasets stored in the 2015 version of the KEGG database [11]. We collected information from the REACTION and COMPOUND datasets from the LIGAND collection. From REACTION, we collected IDs and equations (including coefficients) from 9910 reactions along with ENZYMES and RPAIR data. For RPAIR, we also used RCLASS identifiers and enzyme category information regarding 15,349 entries; we retained only RPairs with RCLASS identifiers. From COMPOUND, we collected the IDs, chemical formulas and molecular weights of 7661 compounds. To limit our analysis to a well-curated and verifiable set, all reactions that included compounds from the GLYCAN dataset and reactions with incompletely described coefficients and subscripts were removed. We also removed 1099 reactions with one compound on each side of the equation from the analysis. Ultimately, 6392 curated reactions were included in the final set.

### Weight metrics

For the balance method, we used molecular weights as reported in the COMPOUND dataset. For the count method, we calculated the frequencies of all Cartesian products and used these numbers as the weight measures.

### Tree structure construction

For every reaction in the dataset, we constructed a TS. We used Perl scripts to construct an algorithm based on the calculated mass differences and frequencies of Cartesian products in the metabolic network to divide each reaction in the dataset. For this purpose, we created two rules, the *balance* and *count rules*.

#### Balance rule

In a given reaction **R**, *A* and *B* are defined as the set of chemical species on the left and right sides of **R**, respectively. Next, we define the operation *P′*(*A*) as the set of A subsets, excluding the empty set.^1^ Elements of the Cartesian product *CP* = *P*^′(*A*)^ × *P*^′(*B*)^ are the basic input of our procedure. For each element (*a*, *b*) ∈ *CP*, excluding the whole reaction, we calculate

1 $$ {d}_{ab}=\frac{1}{K}\left|\left({W}_a-{W}_b\right)\right|, $$

where *K* =  *max* (*W*_*a*_, *W*_*b*_) and *W*_*a*, *b*_ is the sum of the molecular weights for all the compounds in a given *CP* (ECP) element for sides A and B, respectively.

To clarify the operations described above, illustrating our procedure applied to the generic reaction *C* + *D* → *E* + *F* nearly step by step is convenient. Therefore, *P*^′^(*A*) = {{*C*}, {*D*}, {*C*, *D*}} and *P*^′^(*B*) = {{*E*}, {*F*}, {*E*, *F*}}. To be concrete, when considering the ECP ({*C*}, {*E*, *F*}),

${d}_{C, EF}=\frac{1}{K}\left|\left({W}_C-\left({W}_E+{W}_F\right)\right)\right|$, where *W*_*C*_, *W*_*E*_ and *W*_*F*_ are the molecular weights of compounds *C*, *E* and *F,* respectively.

Next, we seek the minimum *d*_*ab*_ among all the elements in the ECP. Because the reaction is balanced, in this case *d*_*whole*_ = 0, we exclude the entire reaction. This ECP with the minimum *d* will be considered the first branch of the TS and will be placed on the right side. In a subsequent step, we eliminate the remaining ECPs containing at least one ECP compound with the minimum *d.* This is an iterative process that continues for each branch until a minimal difference among each ECP cannot be established or no remaining ECPs are found.

#### Count rule

The *count rule* is a means of selecting ECPs based on their occurrence in the metabolic network. This rule was inspired by other works reporting that some compounds are frequently used in metabolic transformations as exchange currency [20–22]. For implementation of this rule, we simply count the frequency of each ECP in the whole network and use this value as a measure to compare compounds within a reaction. When applied, we took the selected branch as the ECP with the highest frequency. The ECP selected with the *balance rule* was placed on the right side, and the remaining ECPs were placed on the left, discarding all ECPs with compounds present in the other group. The *balance rule* is always applied on an ECP first, and the *count rule* is used to disambiguate cases in which the *balance rule* fails to select a single ECP.

#### Reaction in a string format (RSF)

After successive application of the rules, we constructed a representation visualized as a tree (Fig. 3 and Additional file 1: Table S2). We also represented each TS in a JSON format (JavaScript Object Notation) and in two simplified formats. These formats are exemplified below; Eq. 2a gives a generic syntax outline, and Eq. 2b specify reaction R00760.

2a $$ root\Big( balance\left( compound\_ compound\right)\left( compound\_ compound\right) $$

2b $$ root\left( balance\left(C 00095\_C 00085\right)\left(C 00002\_C 00008\right)\right) $$

2c $$ >\left(!\left(C\_C\right)\left(C\_C\right)\right) $$

In Eq. 2c, “>” indicates the tree root for the TS, and “!” indicates the split according to the rule used (one mark (“!”) indicates the *balance rule*, and two marks (“!!”) indicate the *count rule*). The number of parentheses around each pair or loner compound show the depth at which it is nested, indicating the number of partitions employed to construct the tree. “*C*” in Eq. 2c represents compound positions independent of the specific compound.

#### CTS grouping

For each reaction, a TS was proposed, and the architectures found were represented as in Eq. 2c. The TSs available for each reaction were clustered into clusters of TSs (CTSs) according to their topology. Table 1 shows the resultant clusters that were grouped considering the arrangements but not the specific compounds within the reaction.

### Pair validation

We compared the pairs generated by our method with the RPair structures stored in the KEGG REACTION file. For this purpose, we counted the frequency with which a TS-compound pair was equal to an RPair with an RCLASS in the same reaction. We then estimated the posterior probability of successful data distribution versus having a failed pair as follows: Let *θ* be the correspondence of the TS-compund pairs with the RCLASS set. Using Bayesian analysis, we were able to determine the distribution of *θ*. The probability *Ρ*(*θ*| *y*) given *y* coincidences between two datasets out of *n* trials follows a beta distribution. For practical purposes, we gave the expectation value as a summary of the whole distribution. This value is easily calculated using the standard formula $E\left(\theta \right)=\frac{y+1}{n+2}$. Therefore, as in the present cases, when *y*, *n* ≫ 1, this result is very close to the ratio *y*/*n* [23]. Here, the parameters α and β are the number of *hits + 1* and *fails + 1*, respectively. This test was performed for the entire set and for the 22 CTS with at least ten reactions using the *betainv* function in GNU Octave 4.2.1. We represented the distribution with the gnuplot 5.0 program.

### Enrichment of ECN classes in TS patterns

We tested for enrichment of the EC numbers classified into CTSs by our method using a two-sided Fisher’s exact test. Additionally, we controlled the false discovery rate using the Benjamini-Hochberg procedure [24]. We evaluated the strength of the enrichment using the odds ratio. For each possible combination of a given EC category “C” and a particular tree “X”, the odds ratio is defined as *(A/B)/(C/D)*, where *A* is the number of ECs of category “C” classified in tree “X”; *B* is the number of ECs that are not of category “C” classified in tree “X”; *C* is the number of ECs of category “C” that are not in tree “X”; and *D* is the number of ECs that are not of category “C” and are not in tree “X”. We considered only odds ratios with *P values* < 0.05. For this purpose, we extracted EC number(s) related to each reaction from the KEGG database, which corresponded to 4552 ECs distributed at least one time in a reaction. Notably, 1134 of 8957 reactions did not have ECs earmarked in the KEGG database. All the graphical representations were created using R scripts developed in RStudio Version 1.0.136 and edited in Inkscape 0.91.

## Additional file

Additional file 1: **Table S1**. List of the reactions split by only the count rule or by the count rule in some step of division. **Table S2**. Representation of the CTS in a string and tree structure format. The first column, shows the CTS ID; second column represents the reaction split in a string; third column shows the graphical (node-edges), representation. **Figure S1**. CTS vs EC_two_digits comparison by the False Discovery Rate. **Figure S2**. CTS vs EC_three_digits comparison by the False Discovery Rate. (PDF 3439 kb)

## Abbreviations

CTS: Cluster of tree structures
ECN: Enzyme Commission number
ECP: Element of the Cartesian product
EPP: Entirely predicted pair
FP: Failed pair
G3P: D-Glyceraldehyde 3-phosphate
MFP: Mixed-failed pair
MPP: Mixed-positive pair
Pi: Inorganic phosphate
RPair: Reactant pair
RSF: Reaction in a string format
TS: Tree structure
TS-compound pairs: Tree structure-compound pairs

## Footnotes

1 The power of a given set *S* is defined as the set of all S subsets, including the empty set *{}* and *S* itself. Therefore, the operation we performed on *A* and *B* can be described in standard mathematical notation as *P′(S) = P(S) \ {{}}*, where *P(S)* is the power of *S*, and the set difference is indicated by “*\”*.
