# Supplementary material for: Identification of reaction organization patterns that naturally cluster enzymatic transformations
Source: BMC Syst Biol. 2018 May 30;12:63. doi: 10.1186/s12918-018-0583-9 (PMC5977463; doi:10.1186/s12918-018-0583-9)

Table S1. **List of the reactions split by only the count rule or by the count rule in some step of division**

Tree Identifier	Reaction	Applied rule	Detail split of tree Structure represented in one line
CTS-17	R00243	balance/count	root(count(balance(_C00080)(C00025_C00026))(balance(C00001_C00014)(C00003_C00004)))
CTS-17	R00248	balance/count	root(count(balance(_C00080)(C00025_C00026))(balance(C00001_C00014)(C00006_C00005)))
CTS-17	R00365	balance/count	root(count(balance(_C00080)(C00037_C00048))(balance(C00001_C00014)(C00003_C00004)))
CTS-17	R00396	balance/count	root(count(balance(_C00080)(C00041_C00022))(balance(C00001_C00014)(C00003_C00004)))
CTS-17	R00446	balance/count	root(count(balance(_C00080)(C00047_C04076))(balance(C00001_C00014)(C00003_C00004)))
CTS-17	R00581	balance/count	root(count(balance(_C00080)(C00065_C00168))(balance(C00001_C00014)(C00003_C00004)))
CTS-17	R00675	balance/count	root(count(balance(_C00080)(C00078_C00331))(balance(C00001_C00014)(C00003_C00004)))
CTS-17	R00676	balance/count	root(count(balance(_C00080)(C00078_C00331))(balance(C00001_C00014)(C00006_C00005)))
CTS-17	R00688	balance/count	root(count(balance(_C00080)(C00079_C00166))(balance(C00001_C00014)(C00003_C00004)))
CTS-17	R01088	balance/count	root(count(balance(_C00080)(C00123_C00233))(balance(C00001_C00014)(C00003_C00004)))
CTS-17	R01212	balance/count	root(count(balance(_C00080)(C00183_C00141))(balance(C00001_C00014)(C00006_C00005)))
CTS-17	R01434	balance/count	root(count(balance(_C00080)(C00183_C00141))(balance(C00001_C00014)(C00003_C00004)))
CTS-17	R02196	balance/count	root(count(balance(_C00080)(C00407_C00671))(balance(C00001_C00014)(C00003_C00004)))
CTS-17	R02618	balance/count	root(count(balance(_C00080)(C00606_C05527))(balance(C00001_C00014)(C00003_C00004)))
CTS-17	R02755	balance/count	root(count(balance(_C00080)(C00680_C03871))(balance(C00001_C00014)(C00006_C00005)))
CTS-17	R03349	balance/count	root(count(balance(_C00080)(C01186_C03656))(balance(C00001_C00014)(C00003_C00004)))
CTS-17	R04200	balance/count	root(count(balance(_C00080)(C03943_C03341))(balance(C00001_C00014)(C00003_C00004)))
CTS-17	R04201	balance/count	root(count(balance(_C00080)(C03943_C03341))(balance(C00001_C00014)(C00006_C00005)))
CTS-17	R04687	balance/count	root(count(balance(_C00080)(C05161_C05825))(balance(C00001_C00014)(C00003_C00004)))
CTS-17	R04688	balance/count	root(count(balance(_C00080)(C05161_C05825))(balance(C00001_C00014)(C00006_C00005)))
CTS-17	R07164	balance/count	root(count(balance(_C00080)(C00049_C00036))(balance(C00001_C00014)(C00003_C00004)))
CTS-17	R07165	balance/count	root(count(balance(_C00080)(C00049_C00036))(balance(C00001_C00014)(C00006_C00005)))
CTS-17	R08569	balance/count	root(count(balance(_C00080)(C16850_C00944))(balance(C00001_C00014)(C00003_C00004)))
CTS-17	R09830	balance/count	root(count(balance(_C00080)(C00082_C01179))(balance(C00001_C00014)(C00003_C00004)))
CTS-18	R00257	balance/count	root(count(balance(C00064_C00025)(C00857_C00003))(balance(C00001_)(balance(_C00013)(C00002_C00020))))
CTS-18	R00573	balance/count	root(count(balance(C00064_C00025)(C00075_C00063))(balance(C00001_)(balance(_C00009)(C00002_C00008))))
CTS-18	R00578	balance/count	root(count(balance(C00049_C00152)(C00064_C00025))(balance(C00001_)(balance(_C00013)(C00002_C00020))))
CTS-18	R01231	balance/count	root(count(balance(C00064_C00025)(C00655_C00144))(balance(C00001_)(balance(_C00013)(C00002_C00020))))

CTS-18	R04068	balance/count	root(count(balance(C02835_C00013)(C00119_C04437))(balance(C00001_)(balance(_C00009)(C00002_C00008))))
CTS-18	R04463	balance/count	root(count(balance(C00064_C00025)(C04376_C04640))(balance(C00001_)(balance(_C00009)(C00002_C00008))))
CTS-18	R05224	balance/count	root(count(balance(C00064_C00025)(C06399_C06503))(balance(C00001_)(balance(_C00009)(C00002_C00008))))
CTS-18	R05225	balance/count	root(count(balance(C00064_C00025)(C06506_C06507))(balance(C00001_)(balance(_C00009)(C00002_C00008))))
CTS-18	R05815	balance/count	root(count(balance(C00064_C00025)(C05773_C06504))(balance(C00001_)(balance(_C00009)(C00002_C00008))))
CTS-18	R08244	balance/count	root(count(balance(C00064_C00025)(C16618_C16619))(balance(C00001_)(balance(_C00013)(C00002_C00020))))
CTS-18	R08855	balance/count	root(count(balance(C00064_C00025)(C05893_C05894))(balance(C00001_)(balance(_C00009)(C00002_C00008))))
CTS-18	R09195	balance/count	root(count(balance(C00064_C00025)(C00083_C06625))(balance(C00001_)(balance(_C00013)(C00002_C00020))))
CTS-18	R09598	balance/count	root(count(balance(C00064_C00025)(C05773_C19724))(balance(C00001_)(balance(_C00009)(C00002_C00008))))
CTS-18	R09599	balance/count	root(count(balance(C00064_C00025)(C19724_C06504))(balance(C00001_)(balance(_C00009)(C00002_C00008))))
CTS-18	R10767	balance/count	root(count(balance(C00169_C00009)(C00397_C18001))(balance(C00001_)(balance(_C00013)(C00002_C00020))))
CTS-18	R10768	balance/count	root(count(balance(C00169_C00009)(C01822_C20699))(balance(C00001_)(balance(_C00013)(C00002_C00020))))
CTS-22	R00011	balance/count	root(count(C19610_C19611)(balance(C00080_)(C00027_C00001)))
CTS-22	R00078	balance/count	root(count(C14818_C14819)(balance(C00080_)(C00007_C00001)))
CTS-22	R02807	balance/count	root(count(C01319_C00703)(balance(C00080_)(C00006_C00005)))
CTS-22	R02810	balance/count	root(count(C00708_C01382)(balance(C00080_)(C00027_C00001)))
CTS-22	R05218	balance/count	root(count(C06504_C06505)(balance(_C00080)(C01847_C00061)))
CTS-22	R09541	balance/count	root(count(C14818_C14819)(balance(C00080_)(C00006_C00005)))
CTS-22	R10798	balance/count	root(count(C00003_C00004)(balance(_C00080)(C05191_C06162)))
CTS-22	R10799	balance/count	root(count(C00006_C00005)(balance(_C00080)(C05191_C06162)))
CTS-22	R10800	balance/count	root(count(C00003_C00004)(balance(_C00080)(C05194_C06327)))
CTS-22	R10801	balance/count	root(count(C00006_C00005)(balance(_C00080)(C05194_C06327)))
CTS-27	R05735	balance/count	root(count(balance(C00011_)(C00207_C00164))(balance(C00001_)(balance(_C00009)(C00002_C00020))))
CTS-27	R06367	balance/count	root(count(balance(_C00009)(C00002_C00008))(balance(_C00001)(balance(C11924_)(C00010_C11929))))
CTS-27	R06368	balance/count	root(count(balance(_C00013)(C00002_C00020))(balance(_C00001)(balance(C11924_)(C00010_C11929))))
CTS-27	R06396	balance/count	root(count(balance(_C00009)(C00002_C00008))(balance(_C00001)(balance(C11405_)(C00010_C11407))))
CTS-27	R06515	balance/count	root(count(balance(_C00013)(C00002_C00020))(balance(_C00001)(balance(C11405_)(C00010_C11407))))
CTS-28	R00801	balance/count	root(balance(C00001_)(count(_C00031)(C00089_C00095)))
CTS-28	R01194	balance/count	root(balance(C00001_)(count(_C00124)(C01235_C00137)))
CTS-28	R01678	balance/count	root(balance(C00001_)(count(_C00267)(C00243_C00124)))
CTS-28	R02411	balance/count	root(balance(_C00137)(count(C01235_)(C00089_C00492)))

CTS-28	R05767	balance/count	root(balance(C00009_)(count(_C00663)(C00689_C00092)))
CTS-32	R00097	balance/count	root(count(balance(C00080_)(C00003_C00004))(balance(C00001_)(C00541_C00992)))
CTS-32	R00107	balance/count	root(count(balance(_C00001)(C00992_C00541))(balance(_C00080)(C00005_C00006)))
CTS-32	R04940	balance/count	root(count(balance(_C05684)(C05696_C00054))(balance(_C00080)(C00005_C00006)))
CTS-32	R10088	balance/count	root(count(balance(C00014_)(C00117_C00018))(balance(_C00001)(C00118_C00009)))
CTS-35	R03877	balance/count	root(count(balance(_C00080)(balance(C00305_)(C02191_C03516)))(balance(C00001_)(balance(_C00009)(C00002_C00008))))
CTS-35	R05227	balance/count	root(count(balance(_C00080)(balance(C00175_)(C06503_C06504)))(balance(C00001_)(balance(_C00009)(C00002_C00008))))
CTS-35	R10470	balance/count	root(count(balance(_C00014)(balance(_C00011)(C20651_C08057)))(balance(C00001_)(balance(_C00009)(C00002_C00008))))
CTS-36	R02078	balance/count	root(count(balance(C00007_C00001)(C00082_C00822))(C00355_C00355))
CTS-36	R02950	balance/count	root(count(balance(C00007_C00001)(C00811_C16828))(C01197_C01197))
CTS-36	R08826	balance/count	root(count(balance(C00007_C00001)(C08565_C17753))(C08543_C08543))
CTS-37	R09435	balance/count	root(count(balance(_C00697)(C19564_C19565))(C00001_C00001))
CTS-37	R09436	balance/count	root(count(balance(_C00697)(C19581_C19582))(C00001_C00001))
CTS-37	R10518	balance/count	root(count(balance(C00080_)(C00007_C00001))(C00005_C00006))
CTS-43	R00557	balance/count	root(count(balance(C00080_)(C00062_C00327))(balance(balance(_C00533)(C00007_C00001))(C00005_C00006)))
CTS-43	R10106	balance/count	root(count(balance(C00080_)(C00062_C00327))(balance(balance(_C00533)(C00007_C00001))(C00004_C00003)))
CTS-48	R00686	balance/count	root(count(C00079_C02265)(balance(C00001_)(balance(_C00013)(C00002_C00020))))
CTS-48	R10531	balance/count	root(count(C20679_C20679)(balance(C00001_)(balance(_C00009)(C00002_C00008))))
CTS-49	R00789	balance/count	root(count(balance(_C00080)(balance(C00014_)(C00001_C00088)))(C00006_C00005))
CTS-49	R00858	balance/count	root(count(balance(_C00080)(balance(C00283_)(C00001_C00094)))(C00006_C00005))
CTS-56	R01724	balance/count	root(count(balance(_C00080)(balance(C00013_C00253)(C01185_C00119)))(balance(_C00001)(balance(C00009_)(C00008_C00002))))
CTS-63	R10376	balance/count	root(count(C20536_C20542)(count(balance(C00080_)(balance(_C00058)(C00007_C00001)))(C00005_C00006)))
CTS-64	R02518	balance/count	root(count(balance(C00007_C00011)(balance(_C00001)(C00544_C05585)))(balance(_C00080)(C00005_C00006)))
CTS-67	R10203	balance/count	root(count(C11142_C11145)(balance(balance(_C00067)(C00007_C00001))(C01847_C00061)))
CTS-68	R04908	balance/count	root(count(balance(C00007_C00027)(balance(_C00014)(C05638_C05639)))(C00001_C00001))
CTS-69	R10012	balance/count	root(count(C20278_C20279)(balance(C00001_C00014)(C00003_C00004)))
CTS-70	R07175	balance/count	root(balance(_C00080)(count(C00010_C02015)(C00006_C00005)))
CTS-19	R00509	count	root(count(C00002_C00053)(C00224_C00008))
CTS-19	R00700	count	root(count(C00282_C00080)(C00003_C00004))
CTS-19	R00874	count	root(count(C00031_C00198)(C00095_C00794))
CTS-19	R00955	count	root(count(C00446_C00103)(C00029_C00052))

CTS-19	R01641	count	root(count(C00118_C00231)(C05382_C00117))
CTS-19	R02090	count	root(count(C00362_C00361)(C00002_C00008))
CTS-19	R03672	count	root(count(C01693_C05604)(C00355_C00822))
CTS-19	R03725	count	root(count(C00019_C15781)(C01802_C00021))
CTS-19	R04269	count	root(count(C00026_C06030)(C03618_C00025))
CTS-19	R04271	count	root(count(C04081_C03621)(C03622_C03622))
CTS-19	R04297	count	root(count(C01108_C02183)(C03743_C03743))
CTS-19	R04455	count	root(count(C00282_C00080)(C04330_C04377))
CTS-19	R04464	count	root(count(C04377_C04488)(C01080_C00876))
CTS-19	R07181	count	root(count(C00282_C00080)(C00006_C00005))
CTS-71	R01068	count	root(count(_C00111)(C00354_C00118))

Table S2. Representation of the CTS in a string . The first column, shows the CTS ID; second column represents the reaction split in a string; third column shows the graphical (node-edges), representation

ID	Compact String	Topology
1	>(!(C_C)(C_C))	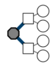
2	>(!(C)(C_C))	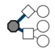
3	>(!(C)(!(C)(C_C)))	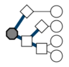
4	>(!(C)(!(C_C)(C_C)))	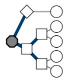
5	>(!(C)(!(!(C)(!(C)(C_C)))(C_C)))	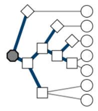
6	>(!(C)(!(!(C)(C_C))(C_C)))	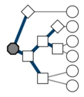

ID	Compact String	Topology
7	>(!(C_C)(!(C_C)(C_C)))	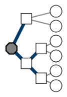
8	>(!(C_C)(!(C)(C_C)))	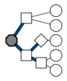
9	>(!(!(C)(C_C))(!(C)(C_C)))	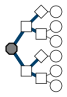
10	>(!(C)(!(!(C_C)(C_C))(C_C)))	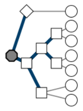
11	>(!(C)(!(!(C_C)(!(C)(C_C)))(C_C)))	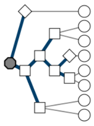
12	>(!(C)(!(C)(!(C)(C_C))))	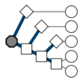

ID	Compact String	Topology
13	>(!(!(C)(C_C))(C_C))	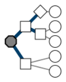
14	>(!(C_C)(!(C)(!(C)(C_C))))	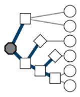
15	>(!(!(C)(!(C)(C_C)))(C_C))	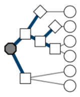
16	>(!(!(C_C)(!(C)(C_C)))(C_C))	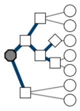
17	>(!!(!(C)(C_C))(!(C_C)(C_C)))	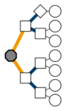
18	>(!!(!(C_C)(C_C))(!(C)(!(C)(C_C))))	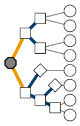

ID	Compact String	Topology
19	>(!!(C_C)(C_C))	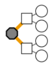
20	>(!(C)(!(C)(!(C)(!(C)(C_C)))))	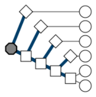
21	>(!(C)(!(C_C)(!(C)(C_C))))	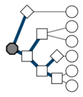
22	>(!!(C_C)(!(C)(C_C)))	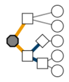
23	>(!(C_C)(!(C_C)(!(C)(C_C))))	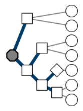
24	>(!(C_C)(!(!(C)(C_C))(C_C)))	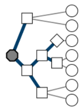

ID	Compact String	Topology
25	>(!(C)(!(!(!(C)(C_C))(C_C))(C_C)))	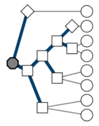
26	>(!(!(C)(C_C))(!(C_C)(C_C)))	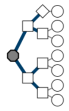
27	>(!!(!(C)(C_C))(!(C)(!(C)(C_C))))	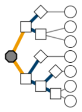
28	>(!(C)(!!(C)(C_C)))	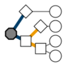
29	>(!(C)(!(!(C)(!(C)(!(C)(C_C))))(C_C)))	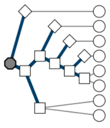
30	>(!(C)(!(!(C)(C_C))(!(C)(C_C))))	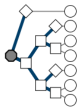

ID	Compact String	Topology
31	>(!(!(C_C)(C_C))(C_C))	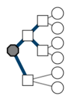
32	>(!!(!(C)(C_C))(!(C)(C_C)))	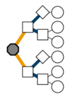
33	>(!(C)(!(!(C)(!(C_C)(!(C)(!(C)(C_C)))))(C_C)))	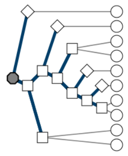
34	>(!(C)(!(!(C)(!(!(C)(C_C))(C_C)))(C_C)))	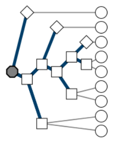
35	>(!!(!(C)(!(C)(C_C)))(!(C)(!(C)(C_C))))	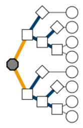
36	>(!!(!(C_C)(C_C))(C_C))	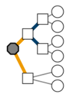

ID	Compact String	Topology
37	>(!!(!(C)(C_C))(C_C))	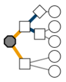
38	>(!(C)(!(!(C)(C_C))(!(!(C)(C_C))(C_C))))	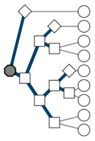
39	>(!(C)(!(C_C)(!(!(C)(C_C))(C_C))))	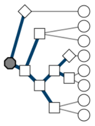
40	>(!(C)(!(!(C)(!(C)(C_C)))(!(C)(C_C))))	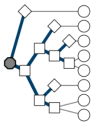
41	>(!(!(C)(C_C))(!(!(C)(C_C))(C_C)))	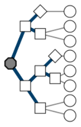
42	>(!(!(C)(!(C)(C_C)))(!(C)(!(C)(C_C))))	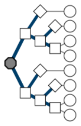

ID	Compact String	Topology
43	>(!!(!(C)(C_C))(!(!(C)(C_C))(C_C)))	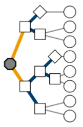
44	>(!(C)(!(C)(!(C)(!(C)(!(C)(C_C))))))	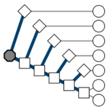
45	>(!(C_C)(!(C)(!(C_C)(C_C))))	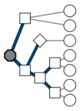
46	>(!(!(C)(!(C)(C_C)))(!(C)(C_C)))	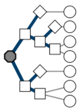
47	>(!(!(C_C)(C_C))(!(C)(C_C)))	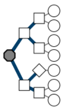
48	>(!!(C_C)(!(C)(!(C)(C_C))))	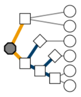

ID	Compact String	Topology
49	>(!!(!(C)(!(C)(C_C)))(C_C))	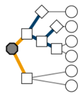
50	>(!(!(C)(!(C)(C_C)))(!(C_C)(!(!(C)(C_C))(C_C))))	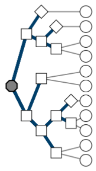
51	>(!(C)(!(!(C)(!(!(C)(C_C))(!(C)(C_C))))(C_C)))	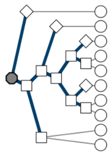
52	>(!(!(C)(!(C)(C_C)))(!(C)(!(C)(!(C)(!(C)(C_C))))))	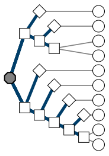
53	>(!(C)(!(!(C)(!(C)(!(C_C)(C_C))))(C_C)))	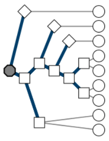
54	>(!(C)(!(!(C_C)(C_C))(!(C)(!(C)(C_C)))))	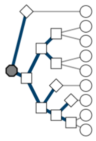

ID	Compact String	Topology
55	>(!(C_C)(!(!(C)(!(C)(C_C)))(!(C)(C_C))))	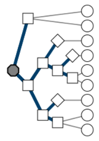
56	>(!!(!(C)(!(C_C)(C_C)))(!(C)(!(C)(C_C))))	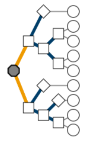
57	>(!(C)(!(C_C)(!(C)(!(C_C)(C_C)))))	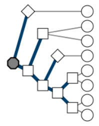
58	>(!(C)(!(!(C)(!(C_C)(C_C)))(C_C)))	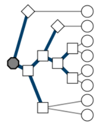
59	>(!(C_C)(!(C)(!(C_C)(!(C)(C_C)))))	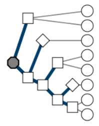
60	>(!(C_C)(!(C_C)(!(C)(!(C)(C_C)))))	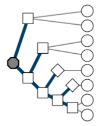

ID	Compact String	Topology
61	>(!(C_C)(!(C_C)(!(C_C)(C_C))))	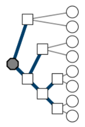
62	>(!(!(C)(C_C))(!(C)(!(C_C)(C_C))))	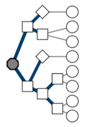
63	>(!!(C_C)(!!(!(C)(!(C)(C_C)))(C_C)))	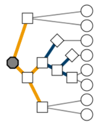
64	>(!!(!(C_C)(!(C)(C_C)))(!(C)(C_C)))	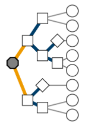
65	>(!(!(C)(C_C))(!(C)(!(C)(C_C))))	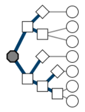
66	>(!(!(C)(!(C)(!(C)(C_C))))(C_C))	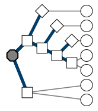

ID	Compact String	Topology
67	>(!!(C_C)(!(!(C)(C_C))(C_C)))	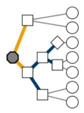
68	>(!!(!(C_C)(!(C)(C_C)))(C_C))	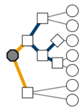
69	>(!!(C_C)(!(C_C)(C_C)))	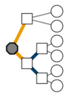
70	>(!(C)(!!(C_C)(C_C)))	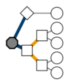
71	>(!!(C)(C_C))	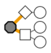

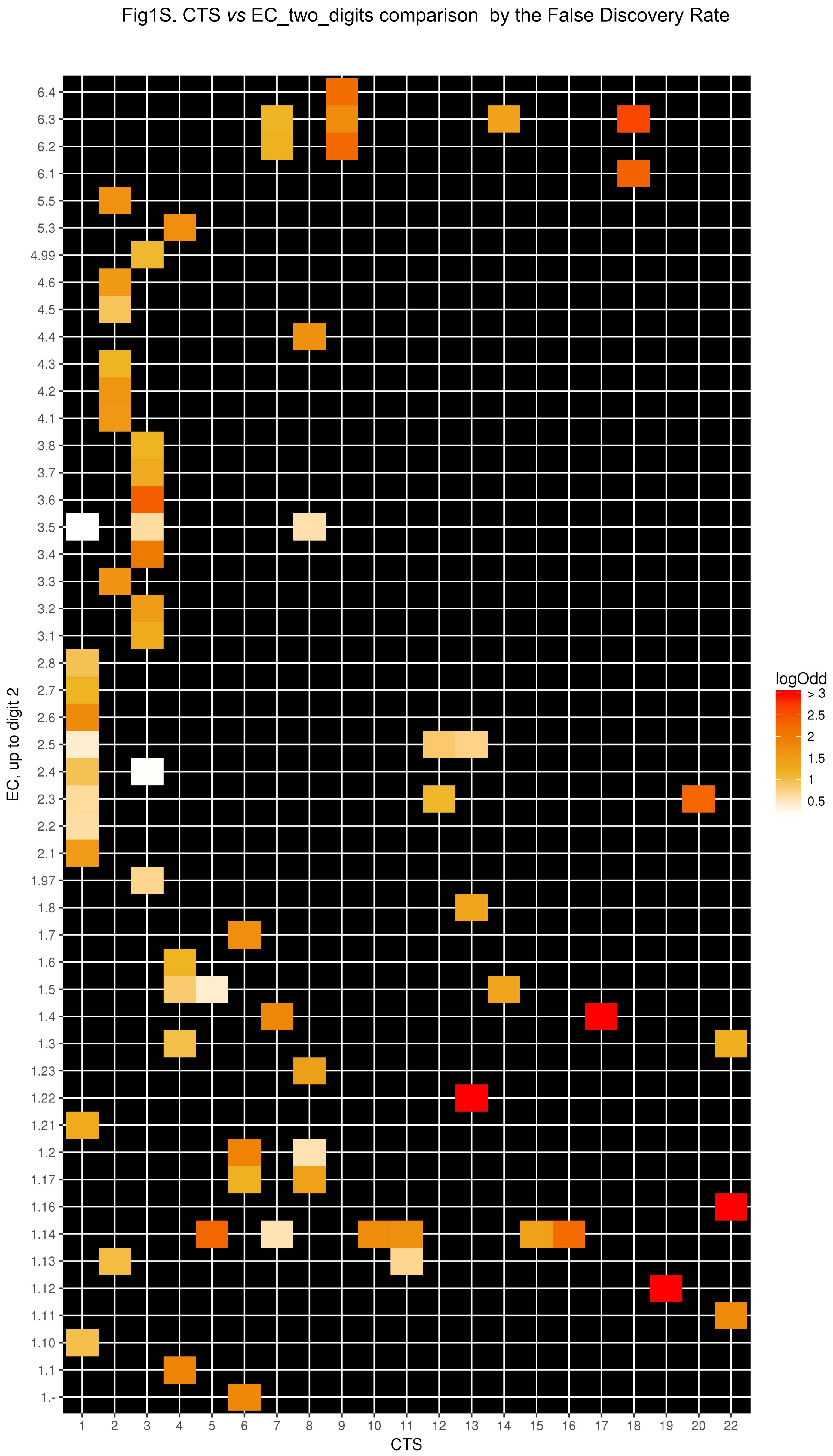
Fig1S. CTS vs EC_two_digits comparison by the False Discovery Rate
EC, up to digit 2
6.4
6.3
6.2
6.1
5.5
5.3
4.99
4.6
4.5
4.4
4.3
4.2
4.1
3.8
3.7
3.6
3.5
3.4
3.3
3.2
3.1
2.8
2.7
2.6
2.5
2.4
2.3
2.2
2.1
1.97
1.8
1.7
1.6
1.5
1.4
1.3
1.23
1.22
1.21
1.2
1.17
1.16
1.14
1.13
1.12
1.11
1.10
1.1
1.-
1 2 3 4 5 6 7 8 9 10 11 12 13 14 15 16 17 18 19 20 22
CTS
logOdd
> 3
2.5
2
1.5
1
0.5

Fig2S. CTS vs EC_three_digits comparison by the False Discovery Rate

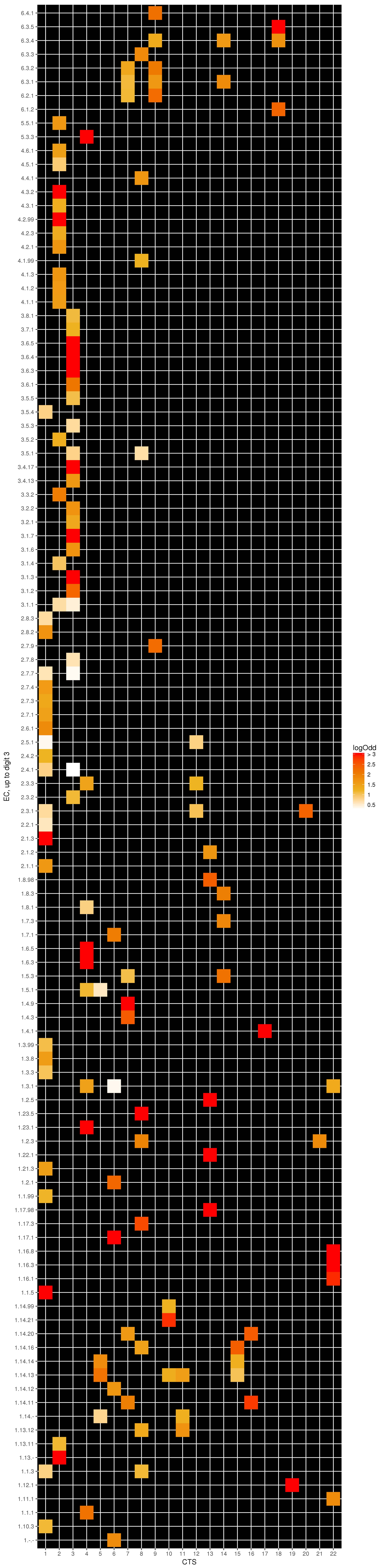

Supplement: Supplementary file 1 — Table S1. List of the reactions split by only the count rule or by the count rule in some step of division. Table S2. Representation of the CTS in a string and tree structure format. The first column, shows the CTS ID; second column represents the reaction split in a string; third column shows the graphical (node-edges), representation. Figure S1. CTS vs EC_two_digits comparison by the False Discovery Rate. Figure S2. CTS vs EC_three_digits comparison by the False Discovery Rate. (PDF 3439 kb) [file 12918_2018_583_MOESM1_ESM.pdf]
